# Highlights lecture EANM 2014: “Gimme gimme gimme those nuclear Super Troupers”

**DOI:** 10.1007/s00259-014-2986-1

**Published:** 2015-02-17

**Authors:** Marion de Jong, Koen Van Laere

**Affiliations:** 1 Departments of Nuclear Medicine and Radiology, Erasmus MC Rotterdam, Rotterdam, The Netherlands; 2 Division of Nuclear Medicine, Department of Imaging and Pathology, University Hospital and KU Leuven, Leuven, Belgium

**Keywords:** Highlights Lecture, 2014, EANM, Gothenburg, Physics and instrumentation, Radiopharmacy, Preclinical imaging, Oncology and radionuclide therapy, Cardiology, Neurosciences, M2M, ISTARD

## Abstract

The EANM Congress 2014 took place in Gothenburg, Sweden, from 18 to 22 October under the presidency of Prof. Wim Oyen, chair of the EANM Scientific Committee. Prof. Peter Gjertsson chaired the Local Organizing Committee, according to the standardized EANM congress structure. The meeting was a highlight for the multidisciplinary community that forms the heart and soul of nuclear medicine; attendance was exceptionally high. In total almost 5,300 participants came to Gothenburg, and 1,397 colleagues participated via the EANM LIVE sessions (http://eanmlive.eanm.org/index.php). Participants from all continents were presented with an excellent programme consisting of symposia, scientific and featured sessions, CME sessions, and plenary lectures. These lectures were devoted to nuclear medicine therapy, hybrid imaging and molecular life sciences. Two tracks were included in the main programme, clustering multi-committee involvement: the 5th International Symposium on Targeted Radionuclide-therapy and Dosimetry (ISTARD) and the first Molecules to Man (M2M) track, an initiative of the EANM Committees for Translational Molecular Imaging, Radiopharmacy and Drug Development. The industry made a substantial contribution to the success of the congress demonstrating the latest technology and innovations in the field. During the closing Highlights Lecture, a selection of the best-rated abstracts was presented including diverse areas of nuclear medicine: physics and instrumentation, radiopharmacy, preclinical imaging, oncology (with a focus on the clinical application of newly developed tracers) and radionuclide therapy, cardiology and neurosciences. This Highlights Lecture could only be a brief summary of the large amount of data presented and discussed during the meeting, which can be found in much greater detail in the congress proceedings book, published as Volume 41, Supplement 2 of the *European Journal of Nuclear Medicine and Molecular Imaging* in October 2014.

## Introduction

The 27th Annual Congress of the European Association of Nuclear Medicine (EANM) took place in Gothenburg, Sweden on 18 – 22 October 2014 under the chairmanship of Prof. Wim Oyen, chair of the EANM Scientific Committee, and Prof. Peter Gjertsson, chair of the Local Organizing Committee. The meeting provided a forum for discussion for those working in the broad field of nuclear medicine in Europe and the rest of the world. The programme reflected the recent progress in this rapidly evolving field. Scientists coming from 82 countries shared their knowledge and presented and discussed new scientific results. The programme was of excellent quality and was based on the standardized EANM congress structure.

The overall programme included plenary sessions, symposia, scientific sessions, featured sessions, poster walks and normal poster sessions, CME sessions and pre-symposia. Two tracks were included in the main programme, clustering multi-committee involvement: the 5th International Symposium on Targeted Radionuclide-therapy and Dosimetry (ISTARD) and the first Molecules to Man (M2M) track, an initiative of the EANM Committees for Translational Molecular Imaging, Radiopharmacy and Drug Development, the latter to promote high-quality research through interaction between basic and translational clinical scientists, stimulating interdisciplinary scientific discussion and educating the clinical scientists in future developments in the field.

**Fig. 1:**
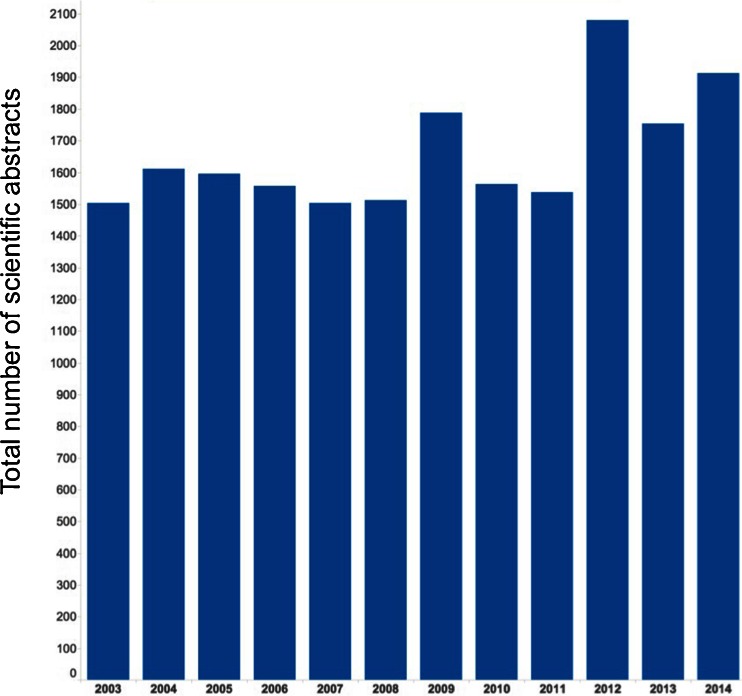
Numbers of accepted scientific abstracts submitted to EANM congresses 2003 – 2014

Original scientific work submitted comprised 1,912 accepted abstracts, along with another 103 technologist abstracts. This was the second highest number of abstracts submitted to EANM congresses (topped only by the Milan 2012 congress with 2,079 accepted scientific abstracts; Fig. 1). The majority of participants came from Europe, while 15.4 % came from Asia and 4.7 % from America.

**Fig. 2:**
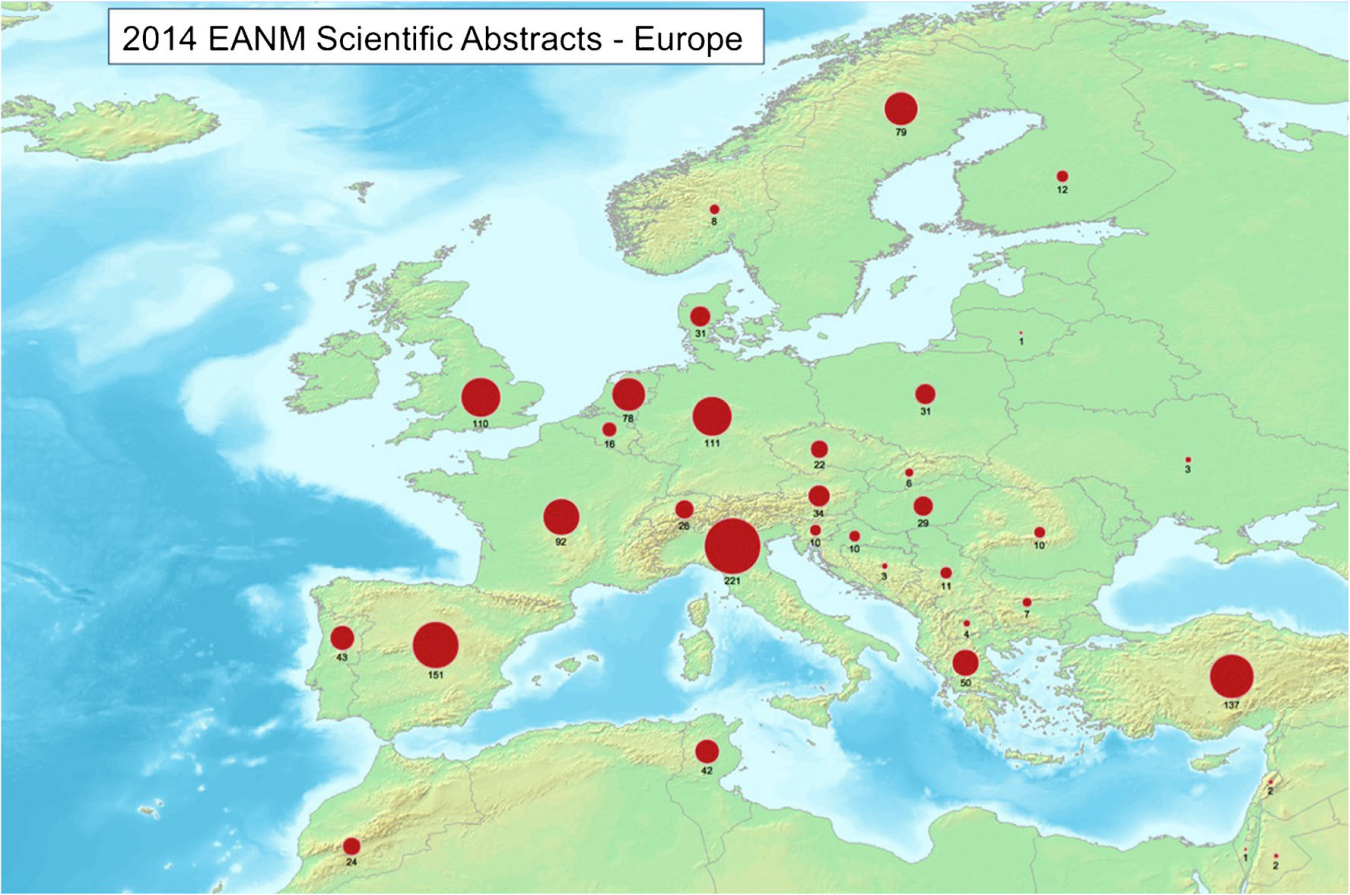
Numbers of scientific abstracts accepted by EANM 2014 per country in Europe

**Fig. 3:**
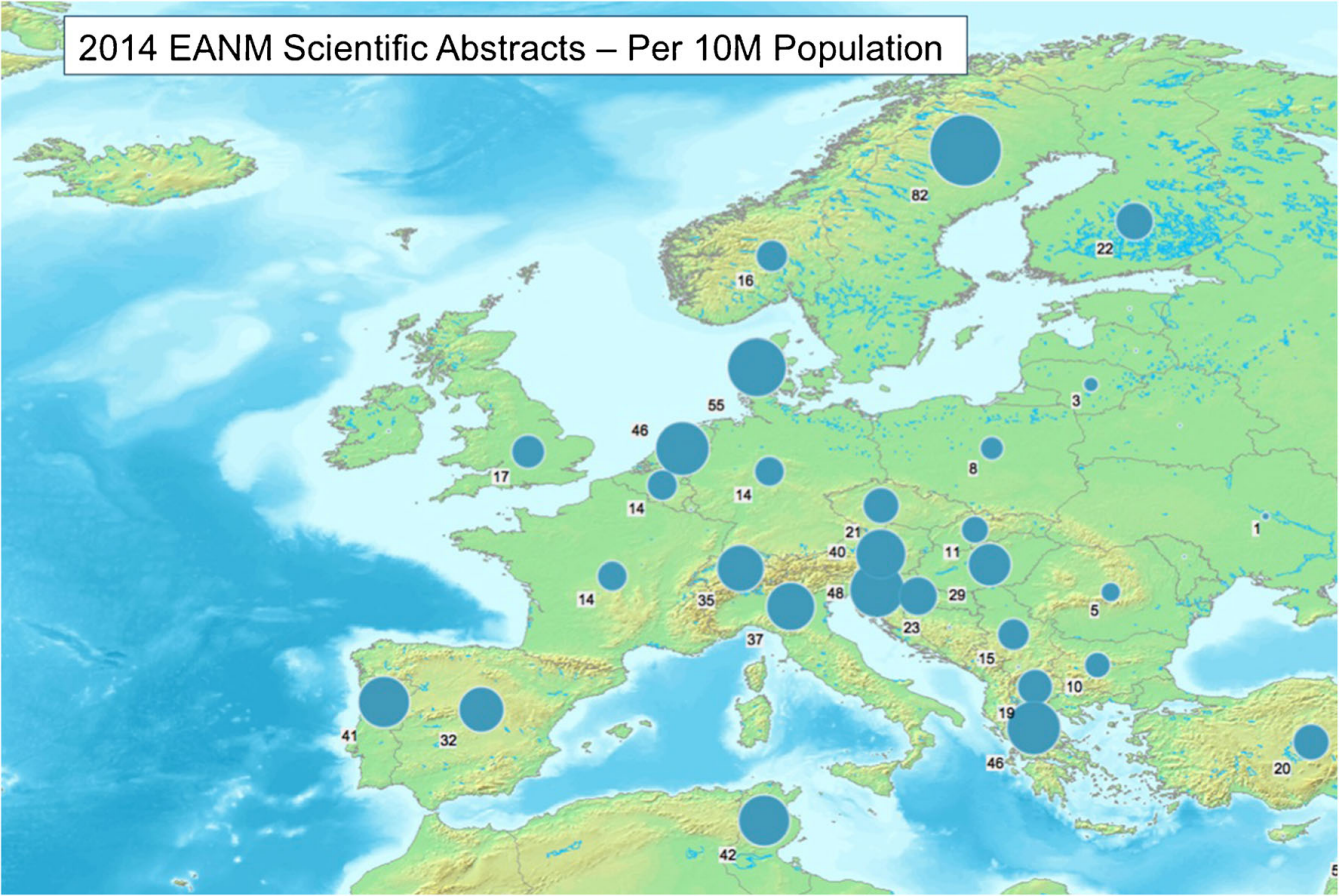
Numbers of scientific abstracts accepted by EANM 2014 per country in Europe, normalized per 10 million inhabitants

**Fig. 4:**
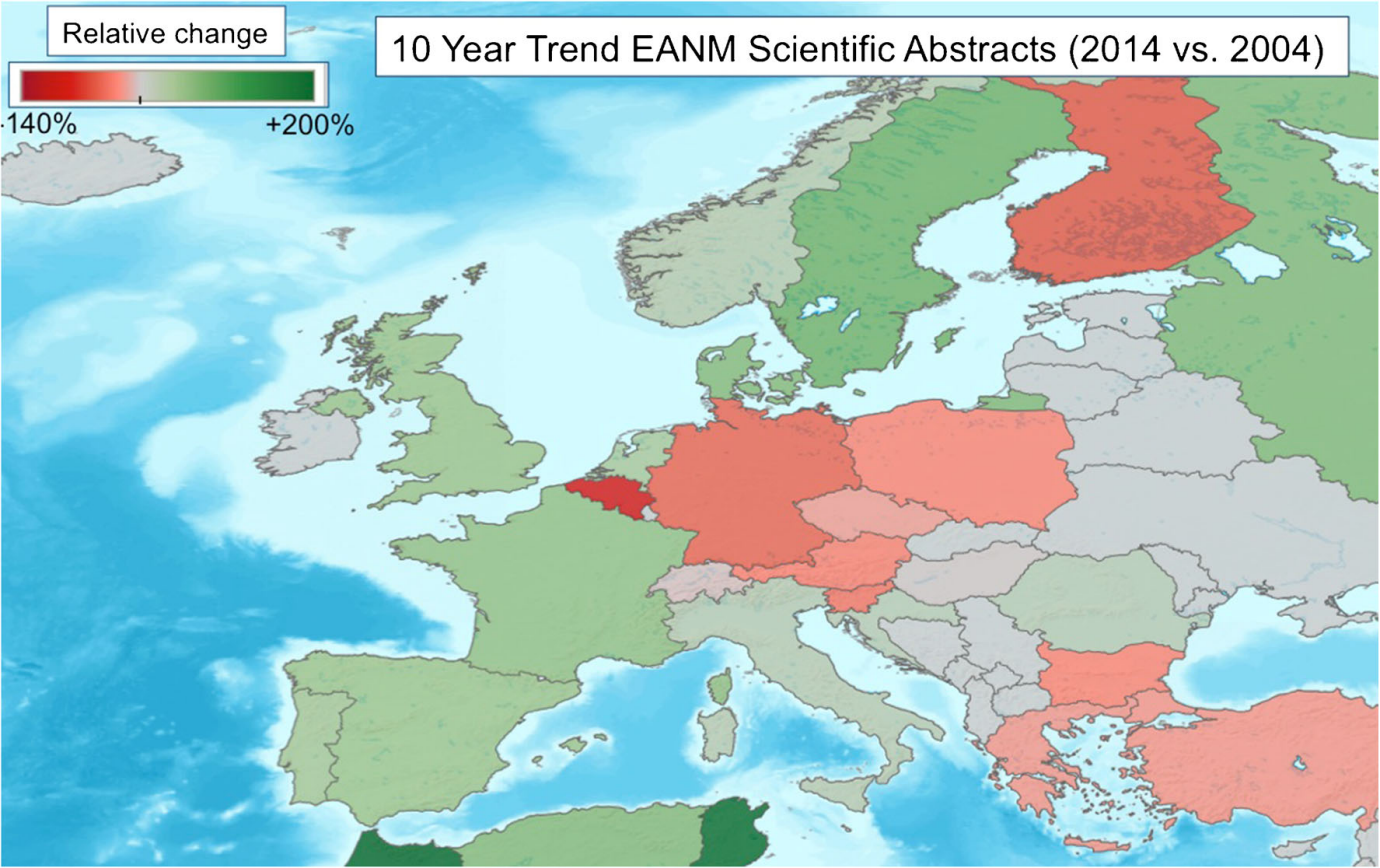
Relative change (%) in number of scientific abstracts accepted by EANM for European countries over the past decade

**Fig. 5:**
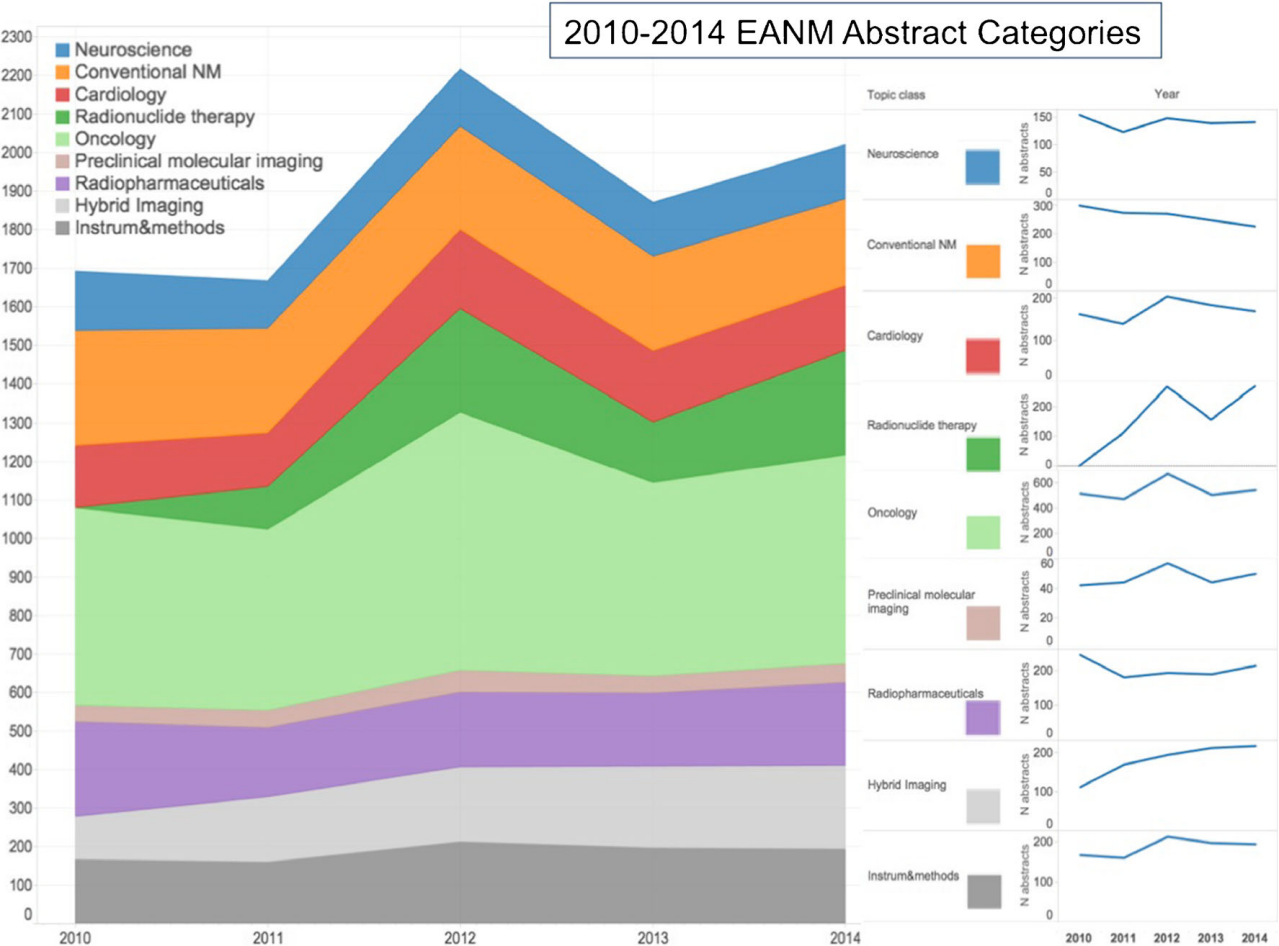
Evolution of abstracts accepted by EANM by category over the past 5 years

Dividing the abstracts by the country of origin of the first author, the major contributing country in absolute numbers was Italy (221), followed by Spain (151) and Turkey (137; Fig. 2). In relative terms per million inhabitants, however, the first place contributor was the host country Sweden (8.2), followed by Denmark (5.5) and Slovenia (4.8; Fig. 3). Of interest, comparing the number of EANM congress scientific abstracts in 2014 with the number ten years earlier (average of 2003 to 2005), an increase was seen for Sweden, Denmark, France, UK and Spain, while other countries (especially Belgium, Finland and Germany) showed decrease in submission rates (Fig. 4). Whether this reflects less domain-specific activity or a dispersion phenomenon through participation in specific clinically oriented or other international specialized imaging congresses remains to be investigated by the national delegates. Dividing the abstracts by category (Fig. 5), the majority of were in the fields of oncology (542, 28 %), radionuclide therapy (270, 14 %), hybrid imaging (217, 11 %) and radiopharmaceuticals (214, 11 %).

**Fig. 6:**
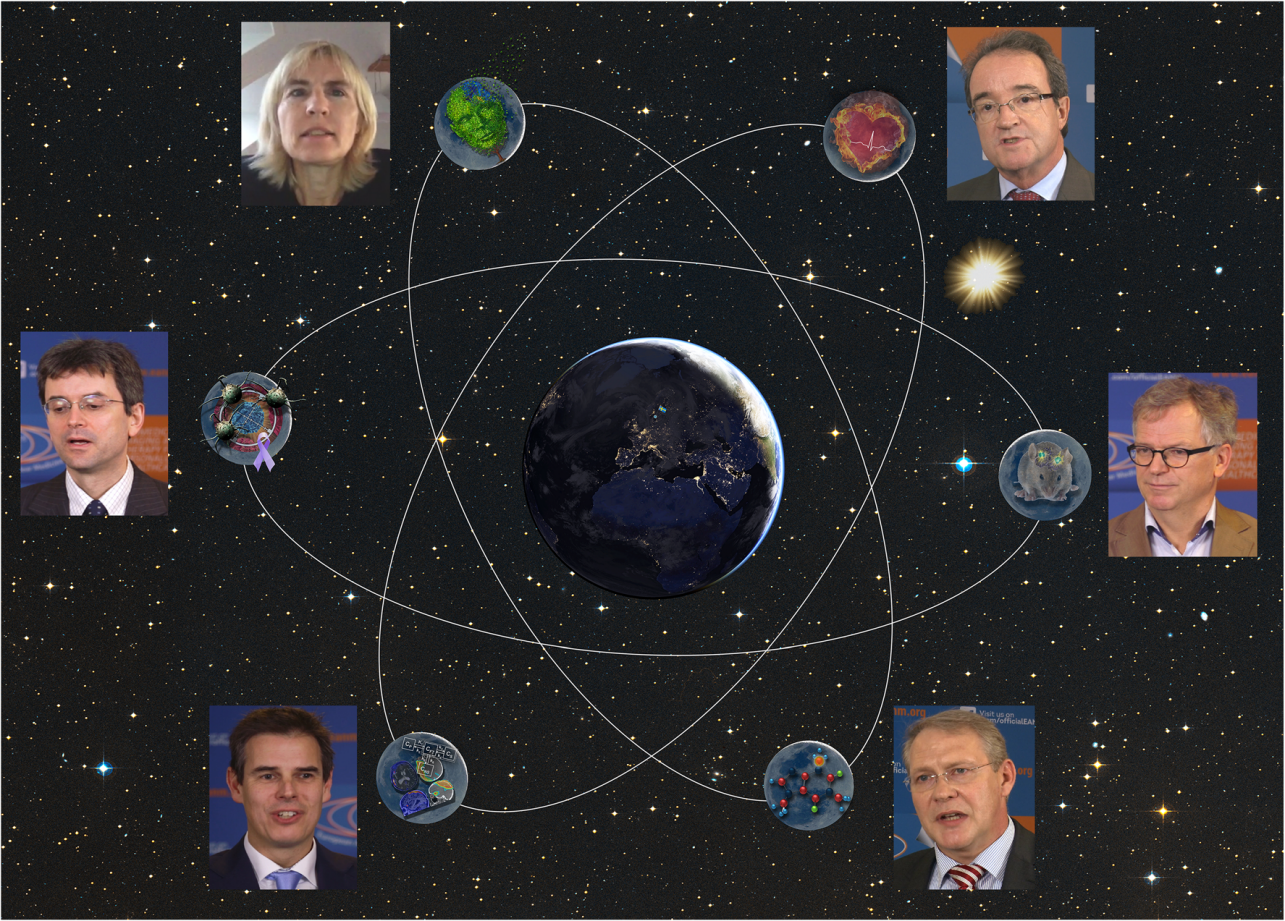
The six nuclear domains depicted in the Highlights Lecture, with the respective experts who were consulted on trends, challenges and developments

All abstracts were graded by expert reviewers and, independent of the topic, the abstracts with the best individual scores were considered for the Highlights Lecture that focused on the following nuclear medicine areas: physics and instrumentation, radiopharmaceuticals, preclinical imaging, oncology and radionuclide therapy, cardiology and brain imaging. For each of these topics, we also invited six European experts (Fig. 6), to share their opinion regarding challenges, novel developments and needs in these specific areas. Their enthusiastic and skilled opinions were also shown in part during the Lecture.

Because of time restrictions, only a selection of the excellent abstracts could be presented during the Highlights Lecture (representing only 40 % of the total presentations submitted for the Lecture). Although strongly guided by reviewer scores, it should be noted that the final inclusion was also based on the personal decisions of the lecturers, seeking to stress important work and developments in the field. We congratulate all authors who were invited to provide slides of their excellent work for possible inclusion in the Highlights Lecture.

## Instrumentation and methods: “Towards hybrid quantitative validation”

**Fig. 7:**
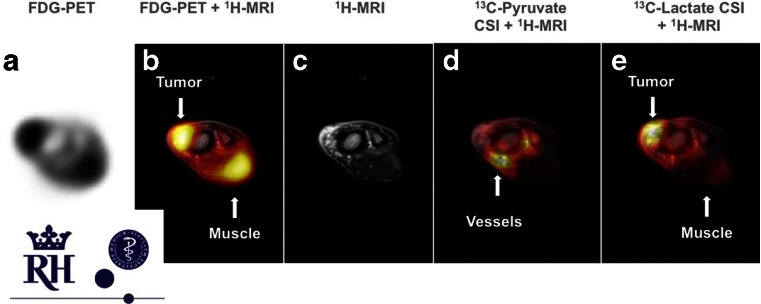
Simultaneous ^18^F-FDG PET and ^13^C-pyruvate PET/MRI imaging of liposarcoma in a dog [1]

One of the most exciting technological developments of the past years is the introduction of the hybrid PET/MRI scanner that combines the high sensitivity molecular imaging with the high time and spatial resolution of MRI. Apart from its structurally superior tissue contrast and resolution, MRI also allows complementary functional and molecular imaging. Gutte et al. [1] used simultaneous PET/MRI with^18^F-FDG and ^13^C-pyruvate in a liposarcoma model, using the dynamic nuclear polarization technique which enables imaging of pyruvate and its metabolites, such as lactate, thereby allowing simultaneous combined imaging of not only glycolysis (FDG), but also increased lactate generation, the well-known Warburg effect. It is expected that this combined technique will allow expanded noninvasive phenotyping of tumours with higher specificity (Fig. 7).

There are a number of important incompletely solved technical challenges with PET/MRI, one of which is undoubtedly the correction for attenuation when using algorithms based on dedicated MR sequences using tissue classification. Boellaard et al. [2] showed that for the different current PET/MRI vendors these algorithms work reasonably well in patients although a quantification bias can exist. Moreover, dedicated acquisition protocols are needed for quality control and multicentre instrumentation calibration, as severe quantitative artefacts and underestimation of SUVs were seen when using clinical protocols for phantom studies. Availability of time-of-flight (TOF) within a simultaneous PET/MRI system may provide an elegant way of estimating joint emission and transmission data.

**Fig. 8:**
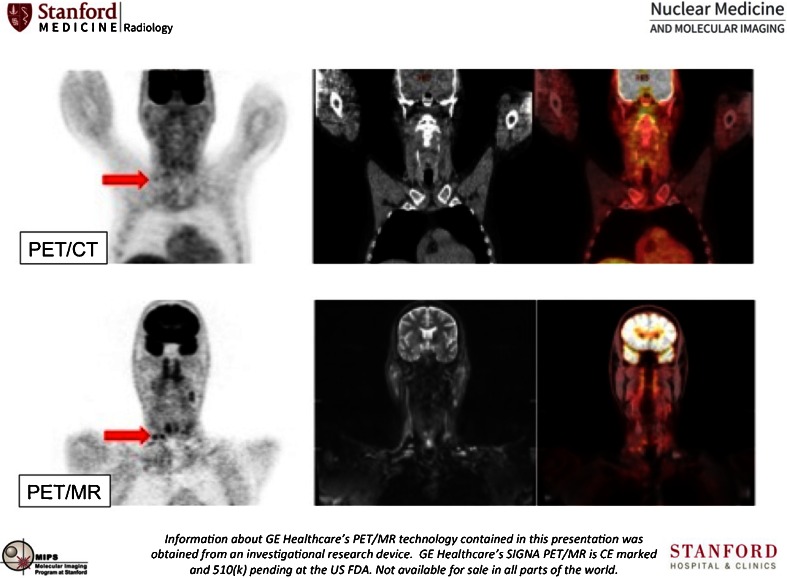
Comparison of TOF PET/CT and TOF PET/MRI for the detection of cervical lymph nodes [3]

**Fig. 9:**
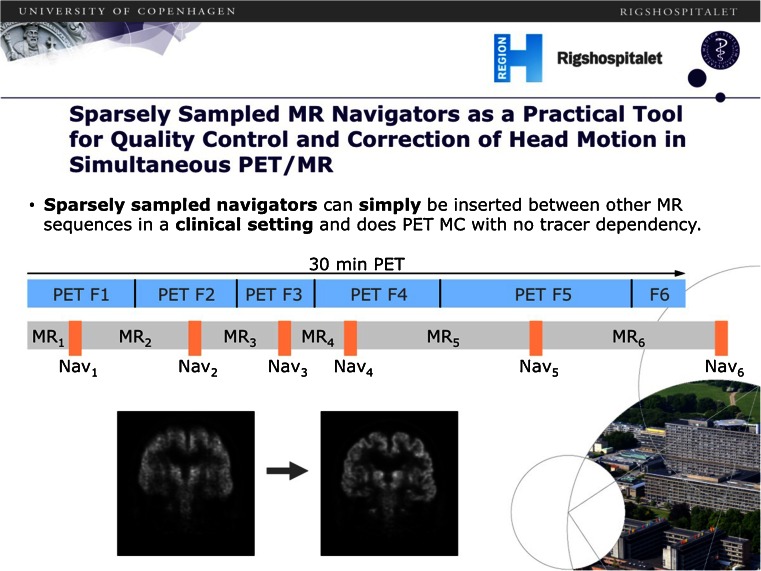
Use of sparsely sampled MR navigators as a tool for correction of head motion in simultaneous PET/MRI [4]

Iaguru et al. [3] reported pilot results of a TOF PET/MRI system in oncology, showing excellent performance of the TOF PET component and examples of improved signal-to-noise and disease detection when compared with a similar TOF PET/CT system (Fig. 8). As in most current comparative studies, PET/MR is performed after PET/CT as the current standard of care technique, which may give rise to some timing bias, so randomized timing studies are needed to further validate these comparisons. PET/MRI can be used for on-the-fly motion correction. Keller et al. [4] showed that sparsely sampled MR structural navigators inside a full MRI multisequence protocol can be used, and results in improved image quality and decreased motion blur (Fig. 9).

Another hardware development for SPECT is the availability of dedicated solid-state cameras for cardiac imaging. In order to realize the ultimate goal of quantitative cardiac SPECT, one important aspect is motion correction, for which solid-state CZT (cadmium zinc telluride) cameras can be sensitive, due to simultaneous multiangle acquisition. Kennedy and Strauss [5] showed that total perfusion defects can be erroneously large with movements that surpass 10 mm. In such cases, selective rejection of projection data where greatest motion induced blur, results in better image contrast and improved perfusion defect quantification. The penalty from lower count rates in the clinical image was marginal because of the high sensitivity of the CZT camera.

In general, PET and SPECT should be quantitative techniques measuring biologically meaningful parameters. Several authors have addressed the important issue of validating simplified approaches compared to full kinetic modelling (allowing better patient comfort or animal welfare in preclinical studies) without introducing significant bias and loss in quantitative accuracy. Alves et al. [6] showed that dual time-point quantification of among others the serotonin synthesis marker ^11^C-5HT-P allows quantitatively accurate measurements without invasive cannulization.

**Fig. 10:**
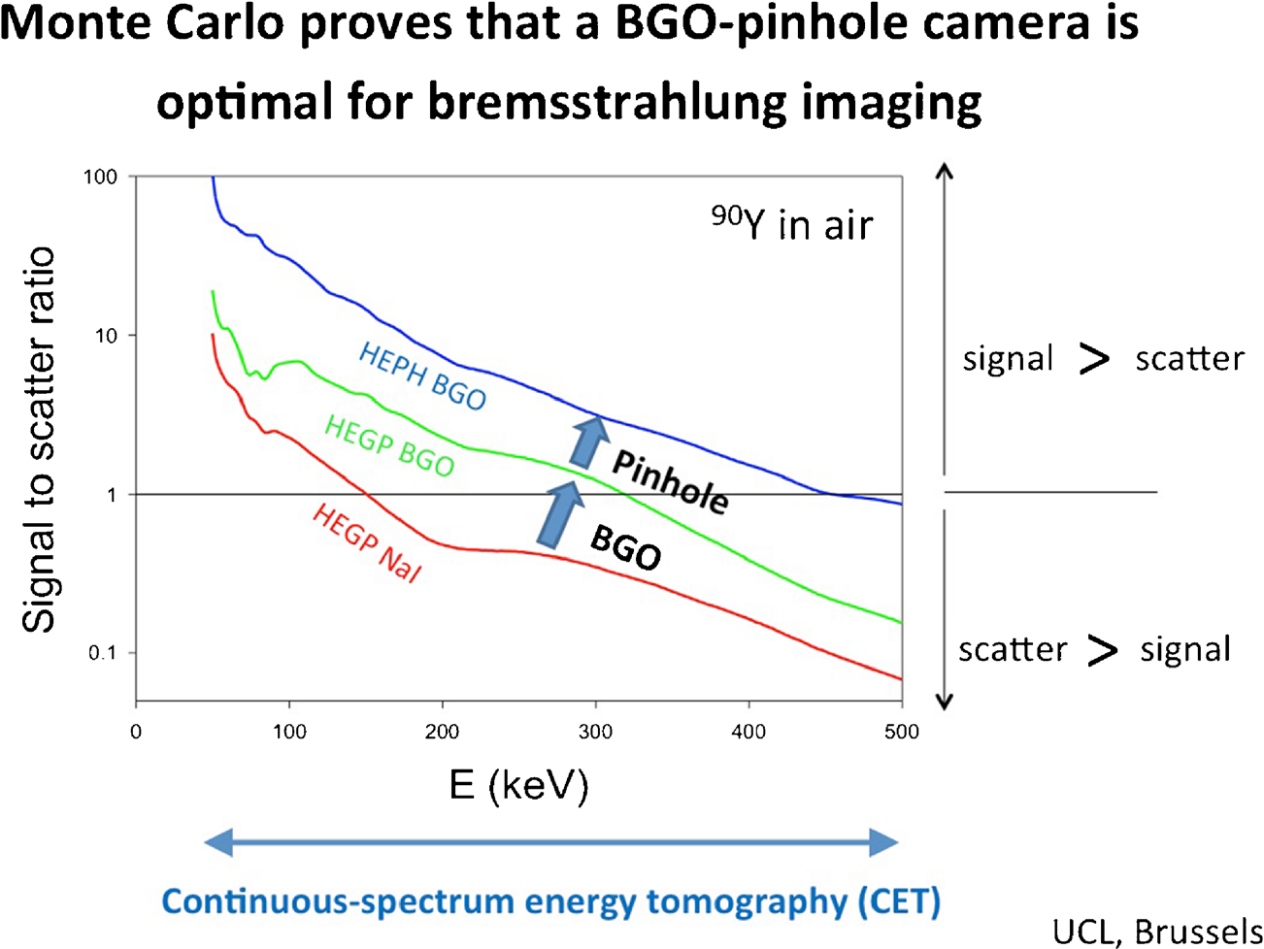
Improved signal-to-noise ratio of BGO detectors for measuring bremsstrahlung in ^90^Y radionuclide therapy monitoring [8]

New radionuclide therapy applications can be monitored using continuous-spectrum bremsstrahlung imaging. Hesse et al. [7] showed that current gamma cameras are not optimal for this goal, as even with a high-energy global purpose collimator, typically only 15 % of detected events are originating from bremsstrahlung as scatter and septal penetration is extremely high. To optimize this, they used Monte Carlo simulations of a new detector with BGO (better stopping power) in combination with a pinhole collimator to ameliorate the signal-to-scatter ratio, resulting in a tenfold higher spectral signal of ^90^Y in air across the whole energy spectrum. This allows fabrication of a portable system for 2-min continuous emission tomography in the catheter room during beta therapy administration (Fig. 10). Finally, as one of the highlighted technologist contributions, Dore et al. [8] showed that conventional dose calibrators for novel radionuclide therapy applications such as ^223^Ra also have the desired linearity and long-term accuracy characteristics for the more challenging physical characteristics of α emitters. They found that low background and absence of contamination traces are even more important in this setting.

We are moving towards quantitation and personalized nuclear medicine, with improvements in dosimetry, standardization and quantification. PET/MRI and CZT SPECT are here to stay, and show considerable promise for addressing true quantitative and multimodal structural plus functional evaluation. To quote Prof. Roland Boellaard (from the movie shown during the Lecture): “The biggest challenge for medical imaging in general is to collect high quality, highly standardized image datasets in order to implement and develop, but also to validate, those new technologies and those advanced data analysis methods”.

## Radiopharmaceuticals and drug development: “Improve the success rates“

A strongly increasing interest in positron-emitting radiometals has been observed recently. In this context ^44^Sc is an interesting candidate. Hernandez et al. [9] explored the advantageous radionuclide characteristics of ^44^Sc for the development of a cyclic dimeric RGD peptide-based tracer for in vivo PET imaging of α_v_β_3_ integrin-associated angiogenesis processes, and using a well-established tumour angiogenesis model showed the feasibility of ^44^Sc for the development of PET tracers.

**Fig. 11:**
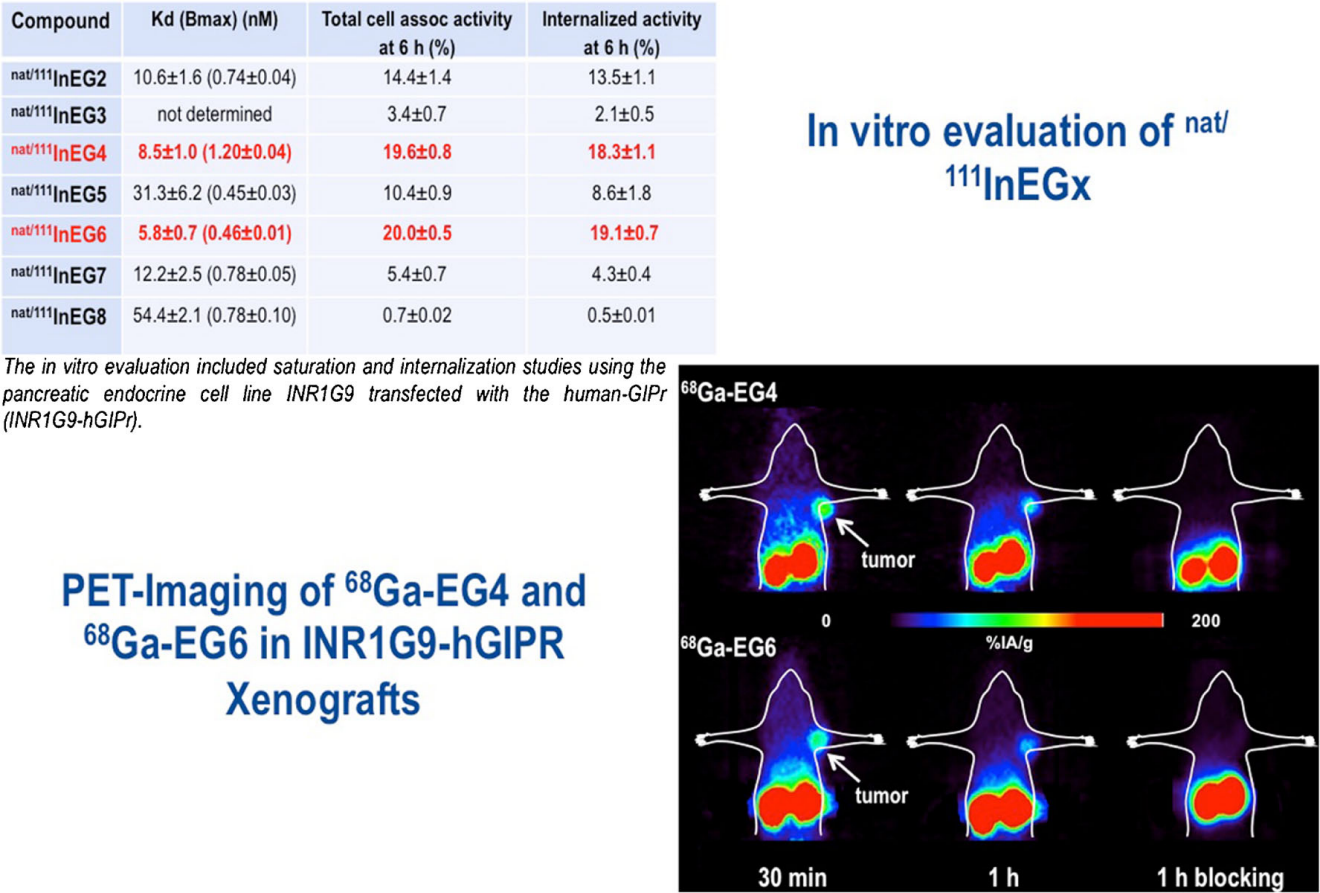
Evaluation of glucose-dependent insulinotropic polypeptide receptor (GIPR) ligands for neuroendocrine tumours [10]

As for new targets, the glucose-dependent insulinotropic polypeptide receptor (GIPR), an interesting target for the imaging of neuroendocrine tumours (NET), was presented by Gourni et al. [10]. This new family of peptide receptors, the incretin receptor family, may be of great importance because it may enable in vivo peptide-based receptor targeting of NETs which do not express somatostatin (sst) receptors, prompting the authors to evaluate newly developed radioligands for the in vivo targeting of GIPR-positive tumours. The good characteristics of several of their newly developed radioligands, based on high receptor affinity, good internalization properties and specific targeting in vivo, support the potential of these radioligands as PET imaging probes for a broader spectrum of NETs (Fig. 11).

Garousi et al. [11] introduced a novel class of scaffold-based affinity proteins, ADAPT (ABD-based derived affinity protein), derived from an albumin-binding domain (ABD). human epidermal growth factor 2-binding (HER2-binding) ^111^In-DOTA-(HE)_3_-ADAPT6 was presented. This protein had promising characteristics in vitro and in vivo in a preclinical model and showed high-contrast in vivo imaging, making it promising for further evaluation, also with short-lived radionuclides.

Prostate-specific membrane antigen (PSMA) is definitely one of the most promising targets for prostate cancer imaging. In this area, there were several very interesting presentations. Nawaz et al. [12] presented a single-chain variable fragment (scFv) of the monoclonal antibody (mAb) J591. A Cys residue was introduced for conjugation with the bifunctional chelator YM103 and labelling with ^68^Ga. The radiolabelled product was stable in serum and showed selective and favourable binding to PSMA-positive cells in vitro and in vivo in mice, warranting further evaluation as a clinical agent for imaging PSMA expression in prostate cancer. Another presentation on PSMA-targeting ligands was given by Chatalic et al. [13]. They developed a novel anti-PSMA nanobody (JVZ-007), a small antibody fragment possessing excellent molecular imaging properties, high target specificity and rapid background clearance. They evaluated the targeting properties of the radiolabelled nanobody in tumour-bearing mice. Replacement of the his-myc tag by the cys tag as well as coinjection with Gelofusine significantly diminished kidney retention, resulting in excellent tumour-to-kidney ratios, bringing the theranostic potential of JVZ-007 in PSMA-positive prostate cancer within reach.

**Fig. 12:**
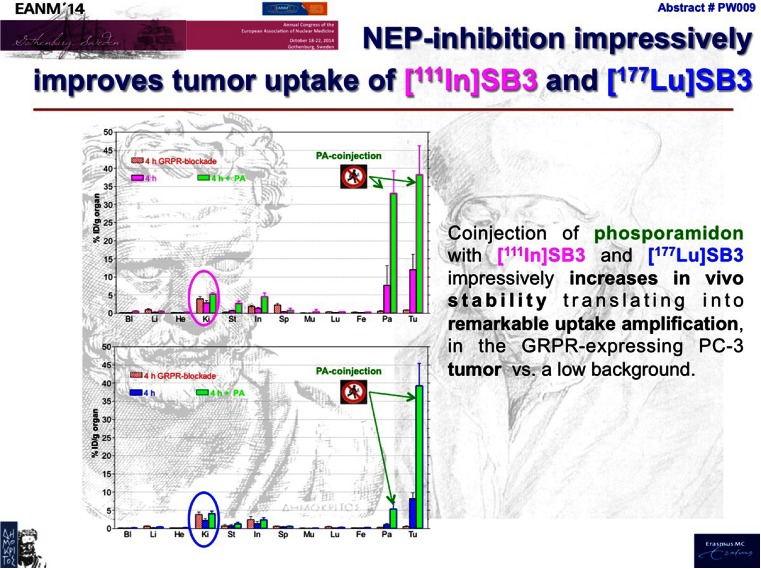
Phosporamidon improves tumour uptake of SB3 [14]

Antagonists targeting gastrin-releasing peptide receptor (GRPR) are also promising for prostate cancer imaging. They offer good tumour retention of radioactivity and fast washout from receptor-positive normal organs without inducing any side effects. Such peptide analogues are however well-known substrates for the protease neutral endopeptidase (NEP) that is abundantly expressed in mammalian tissues. Lymperis et al. [14] introduced SB3, a new DOTA-functionalized analogue of the potent and selective GRPR antagonist [DPhe 6,Leu-NHEt 13]BBN(6-13)], suitable for labelling with the theranostic ^111^In/^177^Lu radionuclide pair. They evaluated the impact of transient in vivo NEP inhibition using the potent, competitive and reversible NEP inhibitor phosporamidon (PA). It was found that PA treatment suppressed the degradation of the radioligands, inducing remarkable enhancement of tumour uptake in a prostate cancer mouse model (Fig. 12). Based on the same principle, Kaloudi et al. [15] explored the impact of PA coinjection with the truncated gastrin analogue [^111^In]SG5 with very high specific activity of ^111^In (by HPLC purification unlabelled SG5 was removed). The combined approach impressively boosted tumour uptake and may allow clinical application of potent internalizing peptide receptor agonists without bioactivity concerns. In vivo NEP inhibition appears to be a very powerful new tool strengthening the theranostic potential of peptide-based radioligands, and further investigation for translation into the clinic is warranted.

The large number of excellent abstracts during the EANM meeting concerning new radiopharmaceuticals, based on new vectors or novel radionuclides, is most promising. Hopes for the future however depend on the successful introduction of such new drugs into clinical use. We quote Prof. Hans-Jürgen Wester (from the movie shown during the Lecture): “To improve our success rate we need goal-oriented alignment of academic research, including the selection of and subsequent focus on the really promising tracers for fast and efficient transfer and clinical assessment based on harmonized protocols”.

## Preclinical imaging: “Pave the translational road“

Preclinical evaluation and imaging studies of many different novel and promising tracers were also presented during the meeting.

**Fig. 13:**
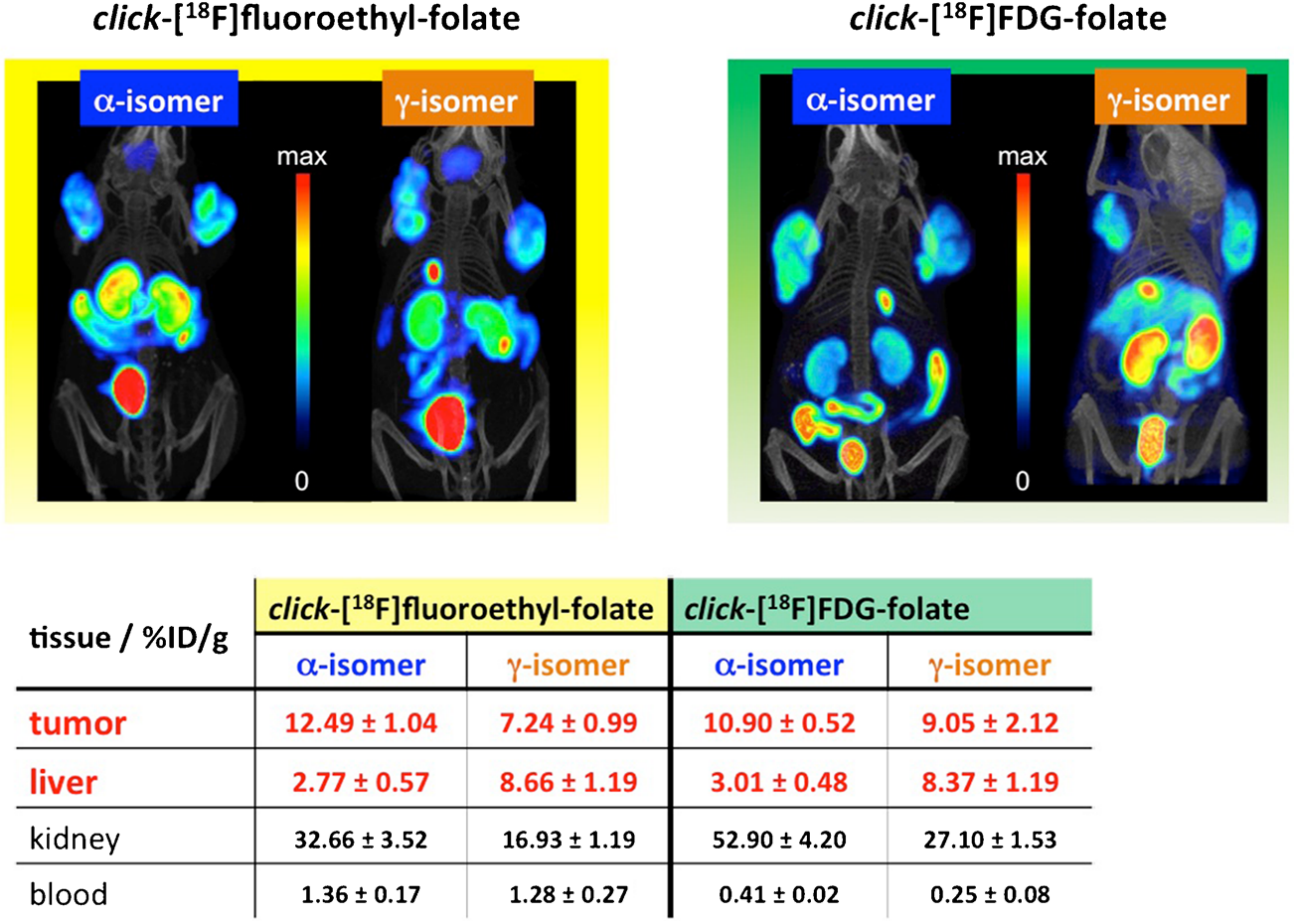
Imaging of α-conjugated and γ-conjugated folate acid derivatives [19]

Due to its association with tumour aggressiveness and metastasis, novel predictive biomarkers associated with epithelial-to-mesenchymal transition (EMT) events in vivo would be very helpful. Hernandez et al. [16] evaluated CD146, an EMT inducer, as a novel in vivo target for noninvasive imaging of EMT in an orthotopic glioblastoma model. They developed a highly specific anti-CD146 mAb (YY146) and presented noninvasive PET imaging of CD146 expression in vivo in mice (Fig. 13), envisaging the potential of this novel mAb for targeted cancer diagnostics and/or therapy.

Radiotracers targeting tumour-associated macrophages (TAMs) also deserve attention. They could be valuable for noninvasive, longitudinal visualization of macrophage infiltration to monitor response of drugs affecting TAMs. Terry et al. [17] developed an imaging agent to monitor TAMs based on the F4/80 receptor, which encodes an adhesion G protein-coupled receptor expressed on the surface of murine macrophages. Binding of ^111^In-anti-F4/80 was specific, but in vivo, ^111^In-F4/80 had a short circulation time as the spleen and liver acted as sink organs. Increasing the antibody dose to 100 μg ^111^In-F4/80 decreased spleen-to-blood and enhanced tumour-to-blood uptake ratios compared to 10 μg ^111^In-F4/80. TAMs could subsequently be successfully visualized by microSPECT, as were macrophages in the spleen and liver.

Amouroux et al. [18] applied targeting of the bradykinin B1 receptor (B1R), a G protein-coupled receptor, which is overexpressed in a variety of cancers but not in normal tissues making it a promising imaging marker for cancer diagnosis. Three ^68^Ga-labelled B1R-targeting peptides generated specific and high-contrast images of hB1R-positive tumour xenografts in mice. P04158 and Z02090 appeared to be the most promising analogues for imaging B1R expression with PET, as they showed significantly higher tumour uptake and target-to-nontarget ratios, probably due to their higher receptor affinity.

**Fig. 14:**
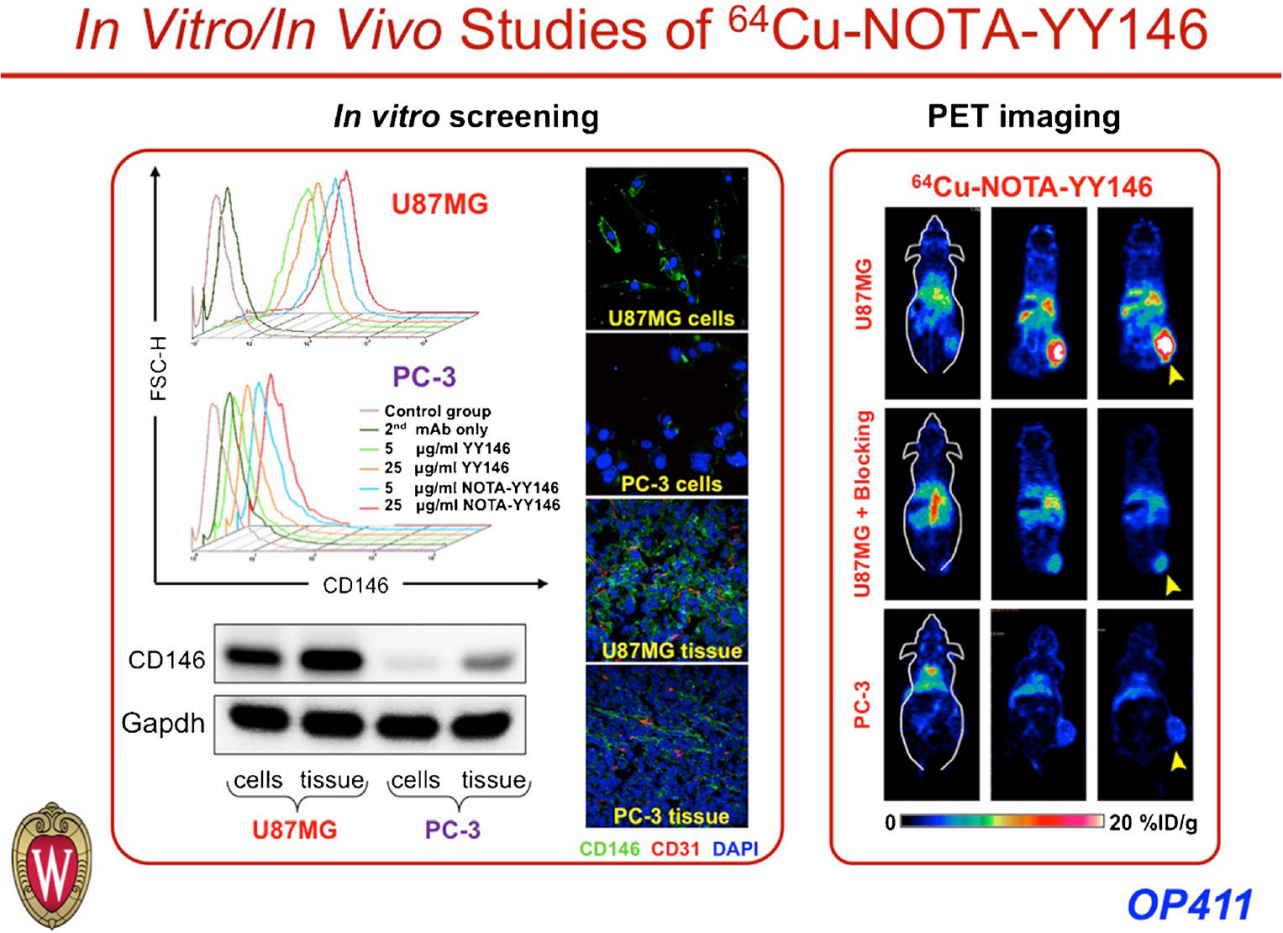
Preclinical studies with YY-146 [16]

The folate receptor is another very interesting target for both imaging and therapy due to its overexpression in a variety of tumour types of epithelial origin. Betzel et al. [19] presented the syntheses, biological evaluation and comparison of two pairs of folic acid conjugates labelled with ^18^F in both the α and γ positions of the carboxylic functionalities. The authors demonstrated that α-conjugated and γ-conjugated folate derivatives show different in vivo properties, while in vitro binding affinity to the folate receptor is almost identical. The site of conjugation on the glutamyl moiety in folic acid can therefore have dramatic effects on the in vivo behaviour of folic acid conjugates (Fig. 14)

**Fig. 15:**
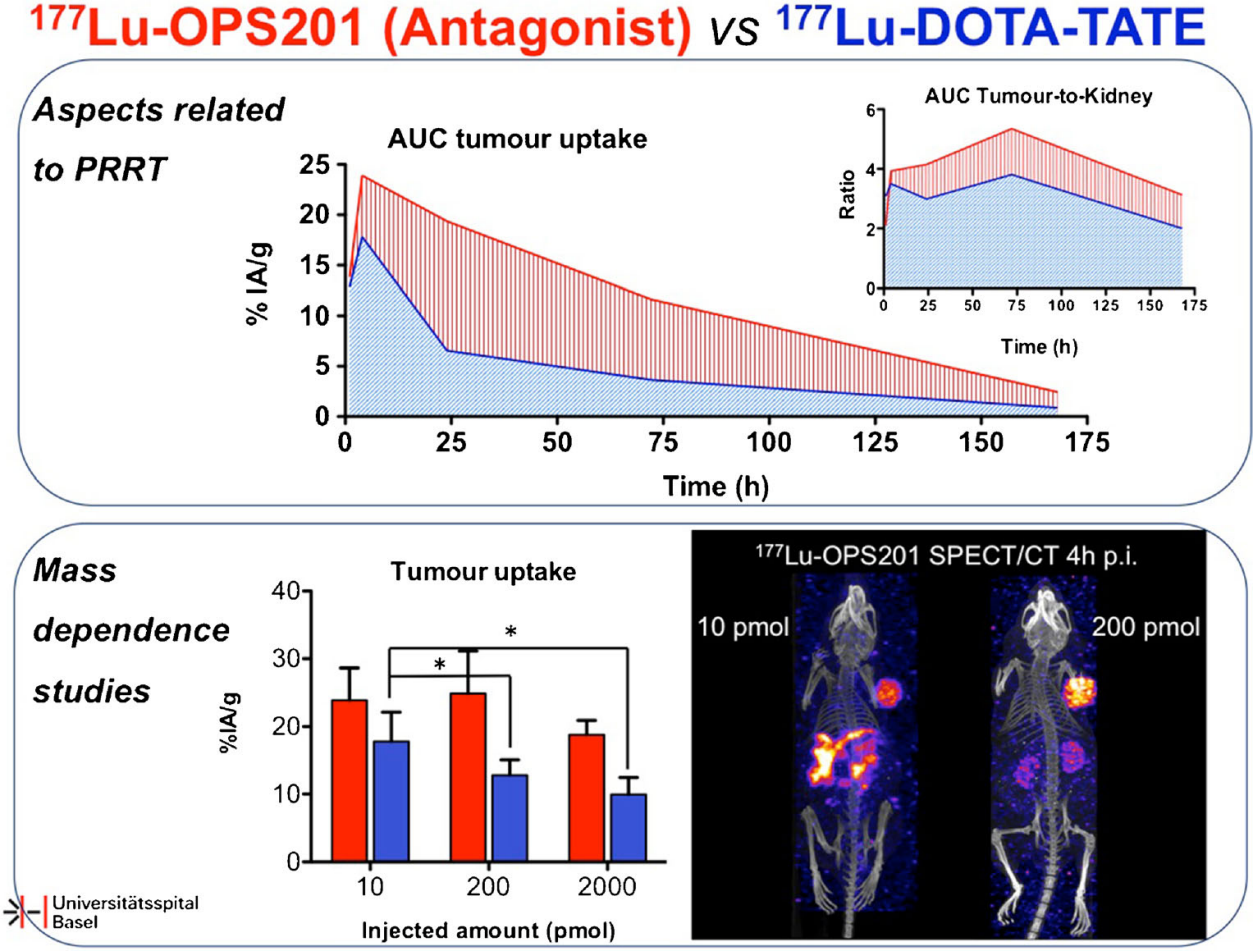
Improved therapeutic index of ^177^Lu-labelled OPS201 versus DOTA-TATE [20]

Very well-known tumour targets are sst receptors. Receptor antagonists have recently been shown to have higher uptake in sst receptor-expressing tumours than sst agonists, possibly due to the higher number of available receptor binding sites. Nicolas et al. [20] evaluated the tumour residence time and therapeutic index of the sst2 antagonist OPS201 compared to the sst2 agonist DOTA-TATE in a theranostic approach in mice. The increased tumour uptake and prolonged residence time of ^177^Lu-OPS201 as well as the favourable differential washout compared to ^177^Lu-DOTA-TATE improved the therapeutic index of the former. The mass-dependent study confirmed the higher number of binding sites for the antagonists in vivo compared to the agonist (Fig. 15).

In several papers the theranostic potential of novel compounds was underlined. Nuclear medicine can offer valuable and clinically important links between imaging and therapy. A highly ranked paper on radionuclide therapy antitumour effects was presented by Yook et al. [21], who aimed to improve localized brachytherapy radiation treatment of locally advanced breast cancer using locally administered EGFR-targeted gold nanoparticles labelled with the β-emitter ^177^Lu (^177^Lu-T-AuNPs). The authors found that the labelled particles were cytotoxic towards breast cancer cells in vitro and slowed tumour growth in vivo. EGFR targeting increased cytotoxic potency in vitro, but appeared not to be required in vivo due to local tumour retention of both targeting and nontargeting particles following intratumoral injection.

Powerful α particle emitters are especially promising for radionuclide therapy, and several papers were presented on this topic. Ladjohounlou et al. [22] compared the anti-tumour efficacy and toxicity of non-internalizing (anti-CEA) and internalizing (anti-HER2) ^212^Pb-labelled mAbs during α-radioimmunotherapy (α-RIT) of small-volume peritoneal carcinomatosis. They also assessed the relationship between tissue absorbed doses and biological endpoints. The authors found a strong therapeutic efficacy of ^212^Pb-labelled mAbs in intraperitoneal α-RIT of small-volume tumours. Internalizing ^212^Pb-labelled mAbs were more efficient, although the tumour absorbed dose was lower than for non-internalizing ^212^Pb-labelled mAbs, which could be explained by the differential distribution: the distribution of the anti-CEA radiolabelled mAb was more heterogeneous at the tumour tissue level.

We hope that the promising tracers and techniques presented here will find their way into the clinic, but several hurdles will have to be overcome. The new M2M Track during the meeting aims to bring together and train translational scientists to improve our successes in this respect. We quote Prof. Otto Boerman (from the movie shown during the Lecture): “Currently it takes too much time and money to determine the potential of useful tracers in the clinic. The micro-dosing guideline is a step in the right direction, but more adjustments are needed. The new M2M Track during the EANM meeting will improve the visibility of translational research in our field and stimulate discussions on how to proceed”.

## Cardiology and vessel disorders: “Harder, Better, Faster, Stronger”

**Fig. 16:**
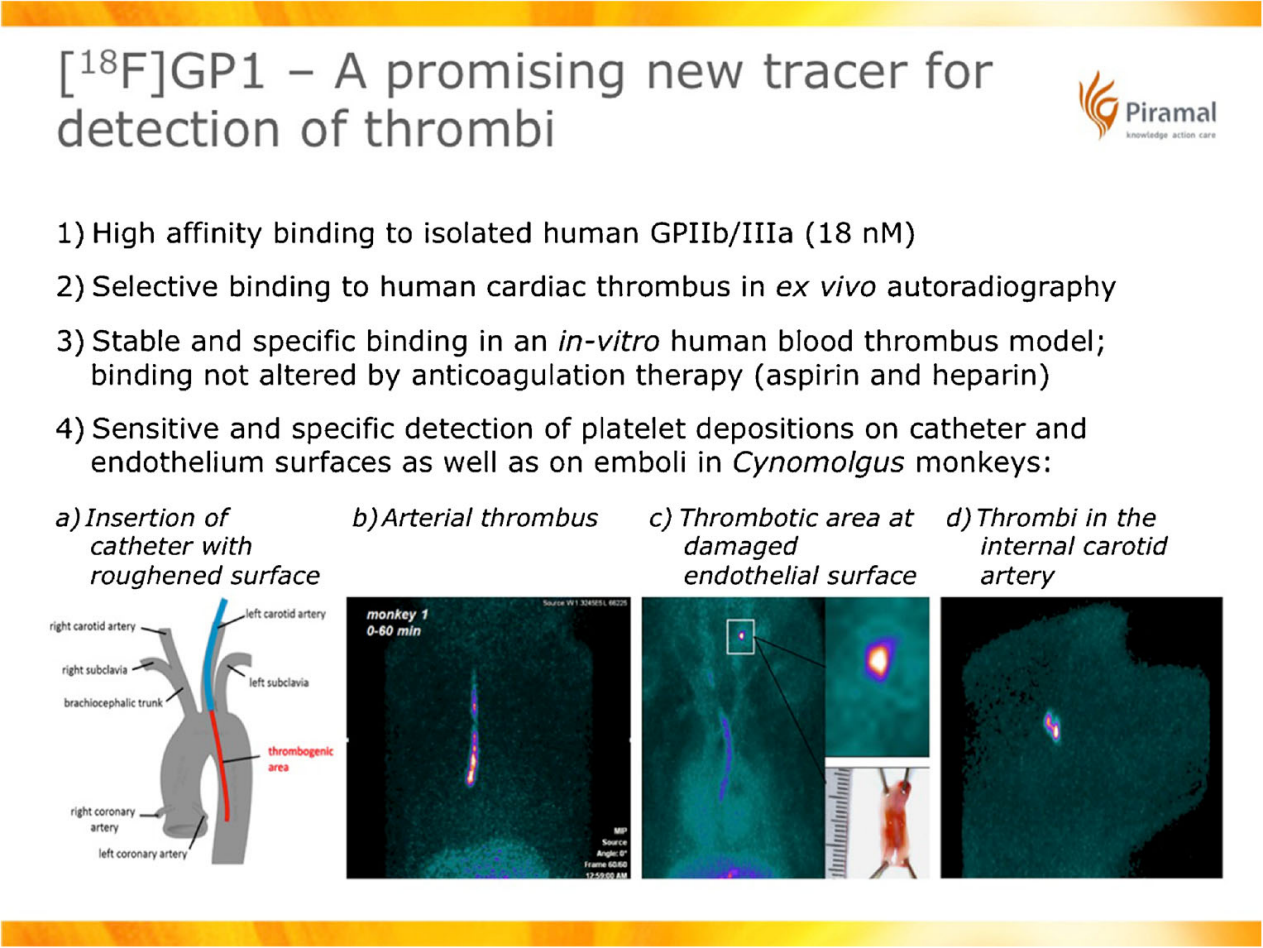
Characterization and preclinical studies of ^18^F-GP1, a new tracer for detection of thrombi [23]

Several new radiotracers that are in late preclinical or early clinical development were presented. Imaging of thromboembolic disease, such as myocardial infarction, stroke, TIA and pulmonary embolism, is of high medical importance as these disorders are major causes of morbidity and mortality worldwide. Currently no established whole-body imaging method is available to systematically search for the embolic source. The glycoprotein (GP) IIb/IIIa receptor is a key receptor required for platelet aggregation by primarily binding fibrinogen and is highly abundant in platelets. Specific imaging of the GPIIb/IIIa was shown for the first time by Stephens et al. [23] in preclinical studies in the monkey in arterial thrombi and on damaged endothelial surface (Fig. 16). The ^18^F-GP1 tracer showed very good imaging characteristics including high affinity (18 nM), in vivo and ex vivo selectivity, stable binding, no influence by anticoagulation therapy and highly sensitive and specific in vivo detection of platelet depositions.

Demeure et al. [24] reported on a novel ^18^F-labelled fatty acid analogue, FCPHA, for imaging of myocardial ischaemia. They showed the results of the phase II trial data assessing safety, kinetics, biodistribution and optimal timing. Diagnostic images can be very rapidly acquired even from 5 min after injection, with no liver interference.

**Fig. 17:**
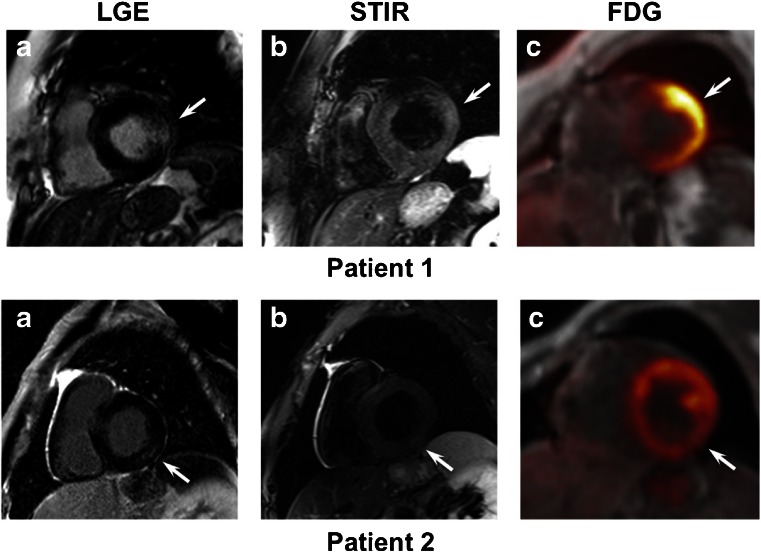
PET/MRI imaging in Anderson-Fabry disease [25]. *LGE* late gadolinium enhancement, *STIR* short inversion time inversion recovery

Also in the domain of cardiology, PET/MRI has promising features and applications. Nappi et al. [25] performed a feasibility study of simultaneous PET/MRI in Anderson-Fabry disease. This X-linked lysosomal storage disorder is associated with severe multiorgan dysfunction and premature death caused by renal failure, cardiomyopathy or cerebrovascular disease. Early diagnosis and comorbidity monitoring is of key importance to assess organ involvement. This group studied 13 patients with normal LV EF and no known cardiac disease, and found complementary information on early disease presence from a combination of late gadolinium enhancement as an expression of intermyocardial fibrosis, short inversion time inversion recovery indicative of oedema, and varying patterns of FDG uptake indicative of inflammation (Fig. 17).

Several novel studies on cardiac imaging systems and methodology were presented. Sciagra et al. [26] used advanced pixel-wise mapping of ammonia PET in patients with hypertrophic cardiomyopathy, in which wall thickness may be large enough compared to the system resolution to study subendocardial ischaemia. After dipyridamole stress, the internal layer was decreased in patients with hypertrophic cardiomyopathy, and the difference after stress was highly significant compared to controls, with also a higher proportion of decreased segments compared to the external myocardial blood flow (MBF). This indicates that pixel-wise parametric mapping could be useful for identifying intramyocardial differences in MBF that are not detectable by conventional segment schemes.

**Fig. 18:**
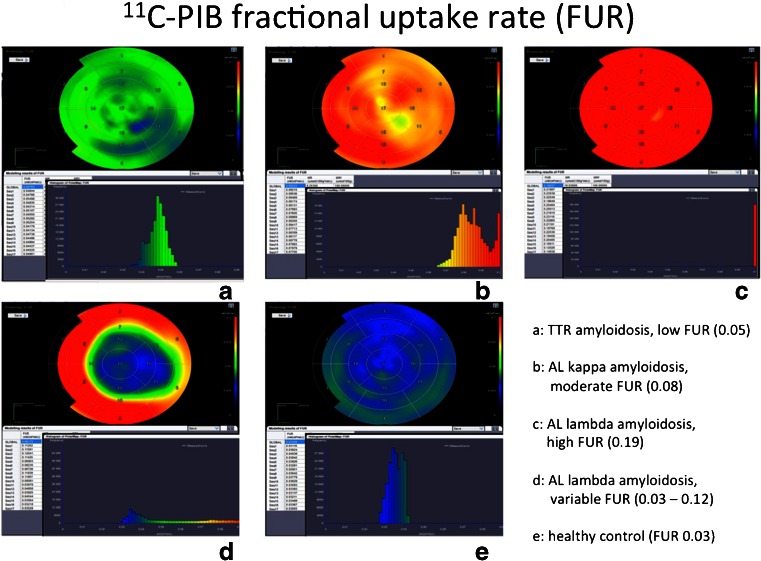
Fractional uptake determination in several forms of cardiac amyloidosis using Carima software [27]. *TTR* transthyretin, *FUR* fractional uptake ratio, *AL* amyloid load

Kero et al. [27] developed an accurate semiautomatic analysis of cardiac ^11^C-PIB retention to allow noninvasive diagnosis of different forms of amyloidosis in the heart. In ten patients with systemic amyloidosis, the cardiac amyloid load was assessed using the fractional uptake ratio as quantitative index. Parametric polar maps and histogram analysis allowed fast, reproducible and straightforward interpretation (Fig. 18), with differentiation of TTR (transthyretin) amyloidosis (with low but increased uptake), light-chain precursor kappa amyloidosis (with moderately elevated uptake) and light-chain lambda fragment amyloidosis (with very high global or regional uptake).

Boemio et al. [28] investigated cardiac insulin resistance, which at the same time represents the cause and the consequence of heart failure and can affect prognosis. These authors investigated whether insulin resistance is associated with impaired cardiac sympathetic innervation in a study with 95 subjects. Insulin-resistant subjects indeed showed impaired heart-to-mediastinum ratios in both the early and late phase, while the washout was not different. Both early and late ratios were related to both fasting insulinaemia and to the HOMA-IR (homeostasis model assessment insulin resistance) index as calculated from fasting glucose and fasting insulin. This relationship gives mechanistic support for the theory that insulin resistance may have an independent adverse effect on prognosis in patients with heart failure through sympathetic nervous system overactivity.

One advantage of recently marketed high-sensitivity dedicated cardiac instrumentation, such as semiconductor cameras, is the dramatic reduction in the combination of activity and acquisition time needed. Perrin et al. [29], who advocate a stress-first examination and perform a rest study only if indicated, showed an average fourfold reduction in effective dose from 14 to 3.5 mSv using CZT SPECT. In a low-risk subgroup with normal stress without a rest study, this was reduced to as low as 2 mSv on average. Despite this dose reduction, they showed in a subgroup of 159 patients using coronarography as the gold standard that sensitivity was retained, implying no loss in diagnostic efficacy.

This congress has shown that in cardiology, we may be able to provide faster and better answers with lower doses and improved technology and with tools to quantify coronary blood flow and reserve, or detect and assess prognosis in specific disorders such as amyloidosis, sarcoidosis, endocarditis and heart failure. We quote Prof. Ignasi Carrio (from the movie shown during the Lecture): “Other new important developments will be PET tracers for the atherosclerotic plaque and eventually also to image the vulnerable ones”.

## Brain imaging: “Chase that dream, together”

In neurodegeneration, enormous strides forward have been made over the past decade, with disease-specific biomarkers, such as amyloid, that have reached the clinic. In addition, several targets have been investigated that go beyond the classical neurotransmitter systems.

**Fig. 19:**
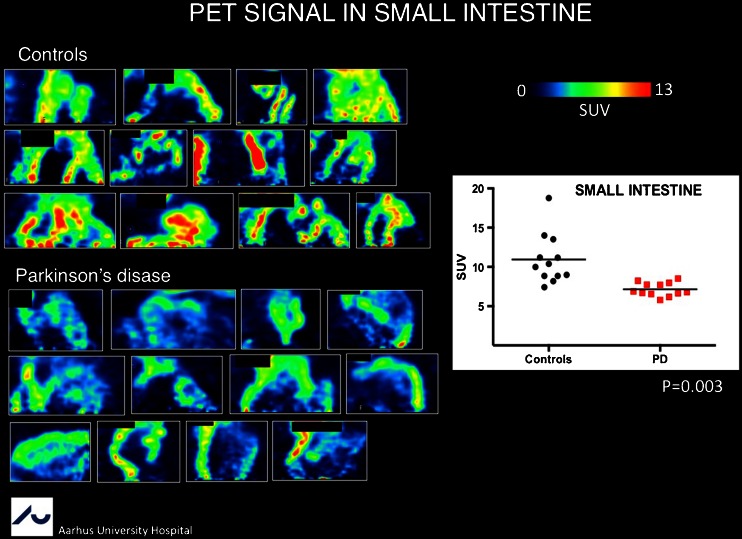
^11^C-Donepezil acetylcholinesterase activity determination in the small intestine in Parkinson’s disease patients and controls [30]

Parkinson’s disease (PD) is not only a movement disorder, but is also characterized by extramotor cerebral abnormalities and extracranial symptoms caused by, for example, parasympathetic denervation inducing vagal nerve pathology. In this year’s Marie Curie Award-winning contribution, Borghammer et al. [30] from Aarhus, Denmark, showed that extracerebral parasympathetic pathology can be visualized using ^11^C-donepezil, which binds to acetylcholinesterase and can be seen as a marker of cholinergic innervation. A strong decrease in the small intestine and pancreas was found in 12 PD patients and with almost only half of the SUV compared to 12 matched healthy controls (Fig. 19). This study is the first showing assessment of peripheral parasympathetic disease involvement in PD.

Fazio et al. [31] reported on a highly specific ^18^F-labelled dopamine transporter (DAT) ligand, PE2I, that has favourable specificity over other transporters, as well as faster kinetics and modelling properties than existing DAT ligands. While up to 70 % loss in patients with early PD occurs in the putamen, high-resolution PET showed that the DAT signal in the substantia nigra (SN) can be quantified, but with much lower abnormality, indicating that the integrity of the SN cell bodies is relatively preserved and that in PD mainly the terminals are affected in the early disease course.

**Fig. 20:**
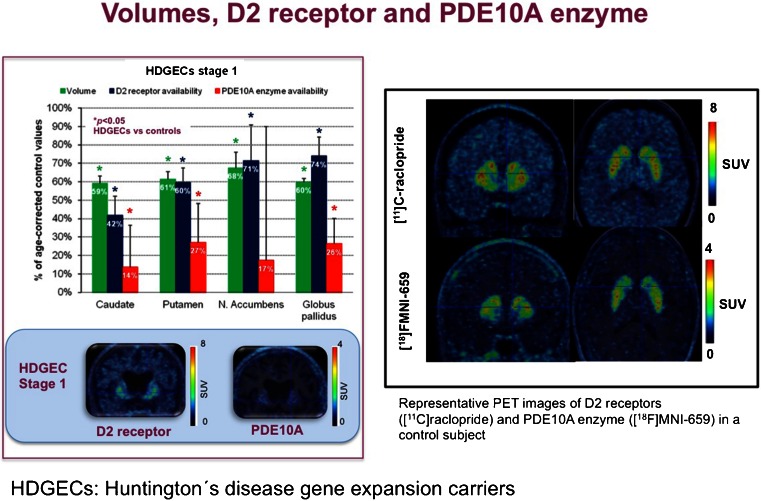
Cerebral phosphodiesterase 10A PET (partial volume-corrected) versus dopamine D2/D3 receptor and volumetric imaging in carriers of Huntington’s disease gene expansion with early-stage disease [32]

Several important modulatory or downstream targets that affect dopaminergic neurotransmission are now under study using molecular imaging. Several tracers targeting phosphodiesterases (PDE), intracellular second messenger systems, were presented at this symposium, including PDE2, PDE4 and PDE10A subtypes which all have high cerebral expression. Fazio et al. [32] used ^18^F-MNI-659, a PDE10A ligand, showing very large changes in patients with early Huntington’s disease (HD) even after partial volume correction. The effect was much larger compared with an only modest D2 receptor change which was previously known as the first abnormal imaging marker for HD (Fig. 20). Studies in presymptomatic HD are being conducted to investigate whether this biomarker may allow disease onset prediction or to provide mechanistic proof for a possible therapeutic target.

The glutamatergic system is another major neurotransmitter system that has not received attention due to lack of appropriate radioligands, but is crucial in many disorders with excitotoxicity (including neurodegeneration, epilepsy, addiction, and schizophrenia). Salabert et al. [33] explored the preclinical validation of ^18^F-FNM, a novel PET tracer for the *N*-methyl-d-aspartate (NMDA) receptor, which is abundantly expressed in cortical and subcortical grey matter. With good blood–brain barrier crossing, fast pharmacokinetics and specific target validation shown by blocking studies, further in vivo human studies with this promising compound are awaited.

Onoe et al. [34] used PET imaging in conscious rhesus monkeys in pharmacological experiments to elucidate a possible antidepressant mechanism of ketamine involving serotonin 1B receptors. Ketamine upregulated the 5HT1B receptor as measured by the novel ligand ^11^C-AZ10419369, only in areas central in the circuitry involved in mood regulation, the accumbens and ventral pallidum. Günther et al. [35] investigated neuroinflammation in rats with highly localized unilateral labyrinthectomy using ^18^F-GE180, a novel TSPO ligand. The authors were able to follow the in vivo time course of the inflammation associated with the initial vestibular syndrome over 30 days that decreased with vestibular compensation. They showed a remarkably strong in vivo signal in the vestibular nuclei that was validated using autoradiography.

**Fig. 21:**
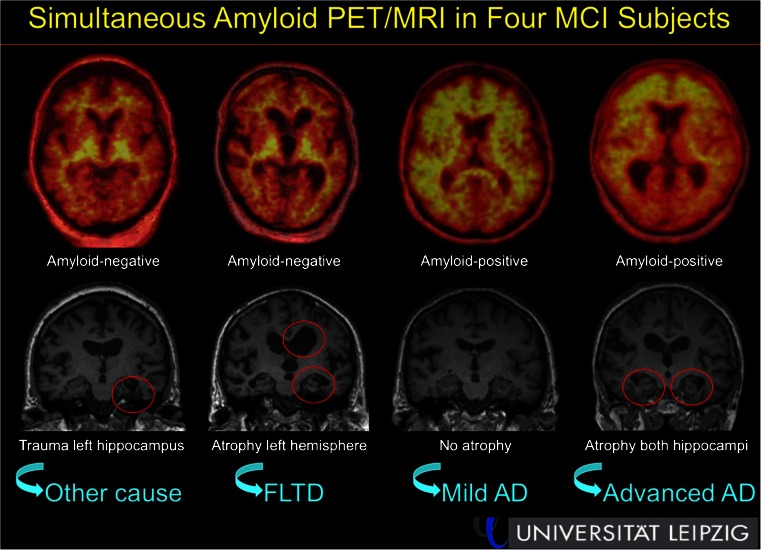
Simultaneous T1-weighted and ^18^F-florbetaben amyloid PET/MRI in MCI patients, allowing improved differential diagnosis and prognosis assessment in a one-stop-shop fashion [36]

Simultaneous PET/MRI sparks high hopes for new research applications in CNS imaging, and this area may also contain the lowest-hanging fruit to allow implementation on a more clinical routine basis. Barthel et al. [36] shared their first experiences with combined amyloid PET/MRI imaging in 100 patients with mild cognitive impairment (MCI) or Alzheimer‘s (AD), and showed that the combination of amyloid and structural imaging of the mesial temporal lobe (as a marker of neuronal injury) significantly improves differential diagnosis and clearly aids the early diagnosis of MCI (Fig. 21).

Pooled post-mortem validation of amyloid imaging shows high sensitivity and good specificity (98 % and 89 %, a negative predictive value of 96 %), confirming that amyloid imaging can reliably exclude amyloid pathology [37]. Maya et al. [38] investigated ^123^I-ABC577 for measuring beta amyloid, and demonstrated good discrimination capacity between AD patients and healthy controls in a pilot study, with the advantage of low nonspecific white matter binding, thus potentially expanding the clinical utility of amyloid imaging using SPECT.

**Fig. 22:**
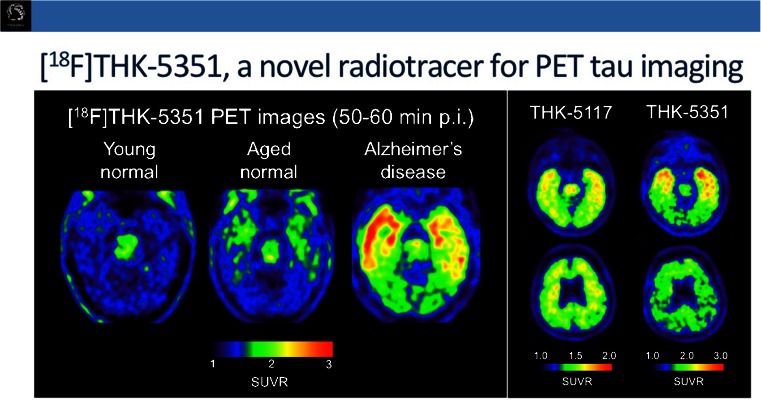
^18^F-THK-5351, a novel radiotracer for PET tau imaging with improved signal-to-noise ratio and lower white matter uptake [40]

However, the field is also moving ‘beyond amyloid’. Imaging trials have shown amyloid to be a very sensitive but very early marker in the disease course of AD, which hampers prediction of disease onset. As tau accumulation probably occurs later in the course of AD in most patients, tau imaging may provide a timelier marker of conversion to overt AD. Moreover, it has potential to characterize the signature of other neurodegenerative disorders that are purely tau-based, such as frontal lobe dementia and progressive supranuclear palsy. Devous et al. [39] provided test–retest data of ^18^F-AV1451 (formerly known as T807) that showed very good reproducibility of 4 – 5 % and high interreader consistency, both advantages necessary for picking up small changes in the early course of the disease and for accurate quantitative evaluation of disease-modifying therapies. Okamura et al. [40] shared the first results in humans from a second-generation tau ligand, ^18^F-THK5351, which has a better signal-to-noise ratio and less white matter uptake than previous Tohuku ligands (Fig. 22).

In general, presentations at the congress on CNS imaging were devoted to developments in tracers, methodology and disease applications. Despite progress in the field of neurodegeneration, there seems to be a worrying decline in contributions in psychiatry. This may in part be reflected by less investment by the pharmaceutical industry in novel targets, since about two-thirds of new radioligands originate in or from collaboration with industry. As molecular imaging remains a unique bridge between genetics and human behaviour, new and collaborative efforts in this exciting area should be stimulated. This was also stressed by Prof. Gitte Moos-Knudsen, whom we quote (from the movie shown during the Lecture): “The main challenges for CNS imaging are the declining number of active programs in pharmaceutical companies resulting in fewer compounds and targets, the cost of PET imaging creating a tendency of underpowered studies, and the need for more and better collaborations not only between imaging centres, but also with basic scientists.”

## Oncology and radionuclide therapy: “Making molecular imaging a clinical reality”

Oncology remains the most important category of abstracts on both diagnostic and therapeutic applications. Many abstracts describing clinical studies on new radiopharmaceuticals were among the highest rated and were therefore selected for inclusion in the Highlights Lecture.

**Fig. 23:**
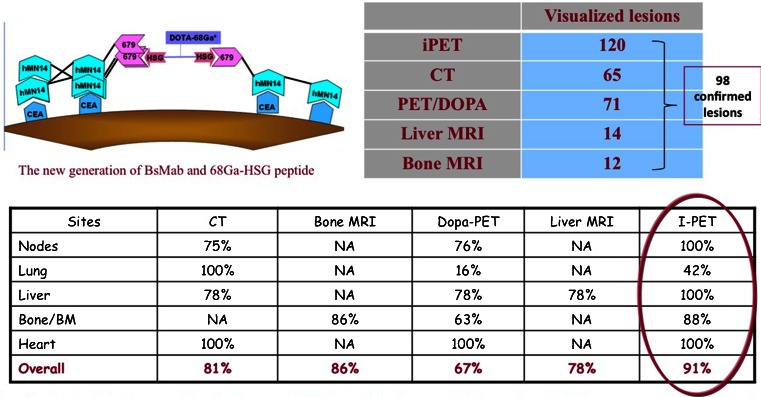
Pretargeted immuno-PET versus CT, MRI and ^18^F-DOPA-PET [42]

Bodet-Milin et al. [41] earlier showed the feasibility of pretargeting using anti-CEA bispecific mAb and radiolabelled hapten peptides. They now compared the sensitivity of whole-body pretargeted immuno-PET/CT and ^18^F-DOPA PET/CT in metastatic patients with medullary thyroid carcinoma (MTC) and demonstrated the potential of anti-CEA pretargeted immuno-PET/CT for staging MTC patients, especially for nodes, liver, and bone evaluation. Pretargeted immuno-PET showed a higher overall sensitivity than CT and ^18^F-DOPA PET/CT, whereas CT seemed to be more effective than immuno-PET/CT for detecting lung metastases. MRI showed better sensitivity than immuno-PET/CT for bone examination, but immuno-PET/CT allowed detection of bone lesions in areas not explored by MRI (Fig. 23).

In the next study, the same group [42] aimed at optimizing molar doses and pretargeting intervals of bispecific mAb and hapten peptide. High tumour uptake and tumour-to-mediastinum blood pool ratios were obtained with pretargeted anti-CEA immuno-PET, especially when using optimized pretargeting parameters: bispecific mAb/peptide molar ratio of 40 and a pretargeting delay of 30 h.

**Fig. 24:**
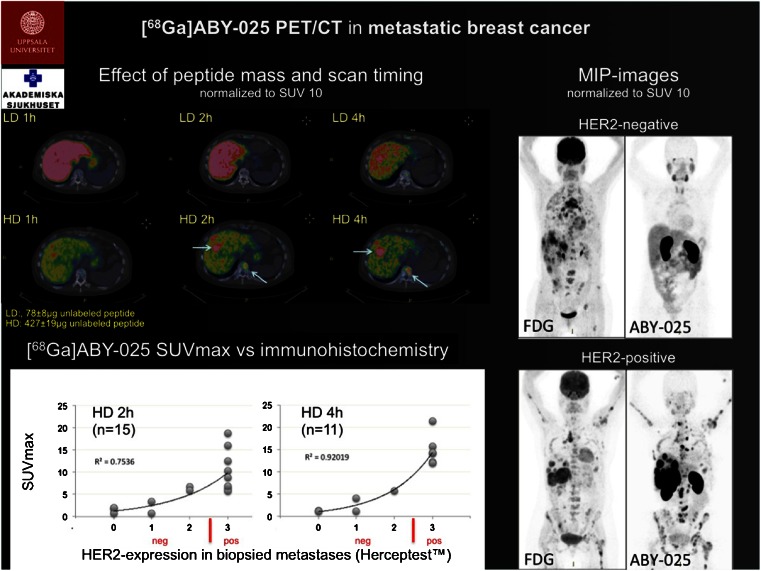
^68^Ga-ABY025 PET/CT imaging in metastatic breast cancer [43]

Another target of interest is HER2, a transmembrane tyrosine kinase receptor overexpressed in 12 – 25 % of breast cancers. Imaging of HER2 enables noninvasive repetitive determination of HER2 status and stratification of patients for HER2-targeted therapy. Sörensen et al. [43] performed a phase I clinical trial of the HER2-binding peptide molecule ABY025 labelled with ^68^Ga for PET to investigate the safety, biodistribution, pharmacokinetics, effect of two peptide masses, and correlation of uptake in tumours with immunohistochemistry. ^68^Ga-ABY025 PET/CT was performed without safety issues and appeared to be a most promising tool for assessing whole-body HER2 receptor status in metastatic breast cancer and imaging with the higher peptide dose (500 μg) at 2 h after injection appeared the best combination for routine use (Fig. 24)

GLP-1-labelled analogues are considered useful tools for visualization of different tumours. Sowa-Staszczak et al. [44] studied the diagnostic efficiency of [Lys40(Ahx-HYNIC-^99m^Tc/EDDA)NH_2_]-exendin-4 scintigraphy. The authors concluded that ^99m^Tc-GLP-1 receptor scintigraphy is a very useful diagnostic tool in patients with clinical symptoms of insulinoma. It enabled the localization of even very small tumours with excellent sensitivity. Moreover, ^99m^Tc-GLP-1 receptor imaging could also be useful in patients with MTC, phaeochromocytoma and gastrinoma, especially when other diagnostic methods are equivocal.

Currently there is huge interest (and also during this meeting) in prostate cancer imaging. KleinJan et al. [45] presented a hybrid tracer, indocyanine green (ICG) ^99m^Tc-nanocolloid, that was earlier found to successfully complement intraoperative radioguidance towards the sentinel node(s) with optical fluorescence identification. For sophisticated procedures such as laparoscopic robot-assisted radical prostatectomy combined with sentinel node biopsy, fluorescence-based identification however required further optimization. They evaluated the effect of tracer preparation, injection technique, and the fluorescence imaging hardware on intraoperative sentinel node identification rate and found a significant improvement in intraoperative fluorescence-assisted sentinel node identification after stepwise optimization of tracer formulation and the fluorescence laparoscope, while preoperative SPECT/CT remained an essential tool for locating the sentinel nodes.

**Fig. 25:**
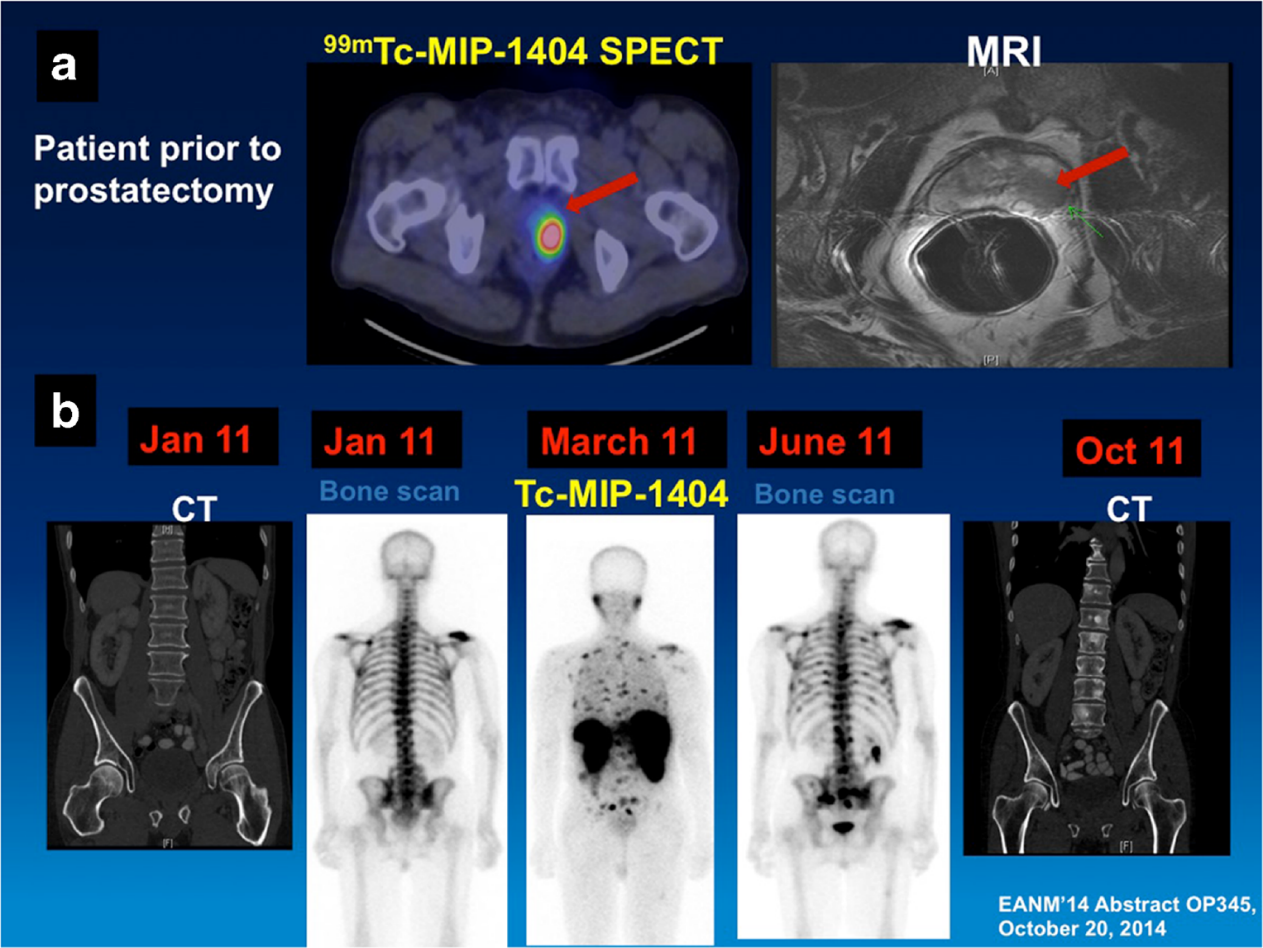
Prostate cancer imaging using ^99m^Tc-MIP-1404 [46]

Prostate cancer cells express different interesting targets for imaging and therapy probes on their membranes. Today's clinical imaging modalities commonly applied to the evaluation of patients with prostate cancer include bone scan, CT and MRI. However, due to the biochemical nature of the disease and its clinical and anatomical course, current imaging techniques have considerable limitations in accurately defining the presence and extent of disease. PSMA is a most promising target for imaging and therapy, and in this area several excellent papers were presented. PSMA is upregulated in aggressive cancers and metastases. Vallabhajosula et al. [46] performed a study using ^99m^Tc-MIP-1404, a small molecule showing specific binding to PSMA (Fig. 25). Compared to bone scan, MRI and CT, PSMA imaging with ^99m^Tc-MIP-1404 detected primary and metastatic prostate cancer in this study with higher specificity. Uptake in the prostate gland lesions correlated well with both Gleason score and PSMA expression. This pilot study clearly documented that PSMA scanning with ^99m^Tc-MIP-1404 may be an appropriate imaging biomarker for early detection of prostate cancer and for selecting patients for therapy.

**Fig. 26:**
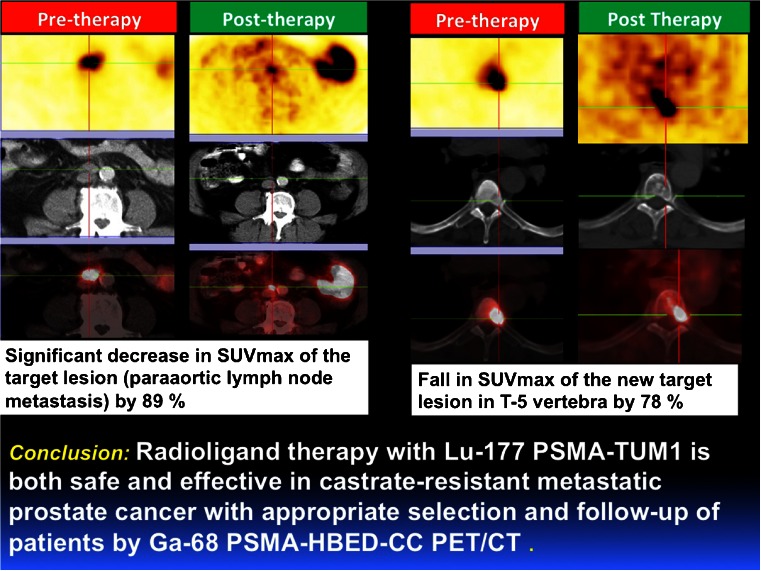
Antitumour efficacy of ^177^Lu-PSMA-TUM1 [48]

 Kulkarni et al. [47] analysed a new PET tracer for PSMA imaging (^68^Ga-PSMA-HBED-CC). The aim of their study was to investigate the role of ^68^Ga-PSMA-HBED-CC PET/CT in patients presenting with rising PSA levels. The high target-to-background ratio enabled accurate detection of very small lesions and of early bone marrow metastases. The authors concluded that ^68^Ga-PSMA-HBED-CC is a very sensitive and specific PET tracer for the detection of prostate cancer with residual/recurrent disease and its metastases. ^68^Ga-PSMA-HBED-CC PET/CT may also be useful for effective stratification of patients undergoing radioligand therapy with ^177^Lu-labelled PSMA ligands. This theranostic approach was also studied by the same authors [48] using EuK-Sub-KFF-DOTAGA (PSMA-TUM1), a promising PSMA-targeting ligand exhibiting high and specific uptake in prostate cancer. The aim of the study was to determine the safety and efficacy of ^177^Lu-PSMA-TUM1 in an initial cohort of patients with castrate-resistant prostate cancer. The treatment was well tolerated by all patients without any significant adverse effects or alterations in any of the laboratory parameters or renal function. The initial results demonstrated that radionuclide therapy with ^177^Lu-PSMA-TUM1 is both safe and effective in castrate-resistant metastatic prostate cancer with appropriate selection and follow-up of patients by ^68^Ga-PSMA-HBED-CC PET/CT (Fig. 26), making PSMA an excellent target for both diagnostic imaging and radiotherapeutic approaches.

Another radionuclide therapy study using a novel tracer was presented by Królicki et al. [49], who investigated a novel treatment strategy for glioblastoma multiforme that is characterized by a very bad prognosis with an overall survival of 9 – 15 months. Substance P, a ligand for the NK-1 receptor system extensively expressed in glioma cells, was labelled with the α emitter ^213^Bi, offering high potential for selective irradiation of tumours while minimizing damage to adjacent tissue. Treatment of patients suffering from recurrent disease with ^213^Bi-substance P appeared safe and was well tolerated. Follow-up of therapeutic responses and toxicity was still on going at the time of the meeting; the median survival of the patients with glioblastoma multiforme was >30 months from initial diagnosis. The authors concluded that targeted α therapy with ^213^Bi-substance P is a promising novel option for treatment of recurrent glioblastoma multiforme.

At this meeting the most promising tracers currently being investigated for clinical imaging and therapeutic applications were discussed. To translate these successes into clinical reality, “we now need to work on efficient and harmonized trial design for validation and dissemination of novel agents and get new radiopharmaceuticals FDA/EMA approved and reimbursed for clinical use” (quote Prof. Wolfgang Weber from the movie shown during the Lecture).

## Conclusion

Nuclear medicine has benefited from rapid technological evolution that has yielded new imaging possibilities, new hardware and software developments and new radiopharmaceuticals which have gained widespread acceptance. The combination of diagnostic imaging, tissue characterization, function measurement and targeted therapy in nuclear medicine remains very powerful. The EANM 2014 Congress provided a sample of such novel nuclear medicine contributions with excellent innovations in (pre)clinical imaging methodology, physics, radiopharmaceuticals and radiochemistry. Novel radionuclide applications in both diagnosis and therapy were presented. Significant progress was demonstrated in the clinical applications of existing nuclear medicine procedures, whereas new applications are under development in preclinical and early clinical stages.

We believe that the achievements of the Gothenburg EANM Congress are symbolized by the renowned international success of the group ABBA, who won the European Song Contest in 1974, and who’s member Björn (Ulvaeus) was born in Gothenburg. The “Super Trouper” nuclear medicine studies presented at the congress, spanning multiple application domains, demonstrated again the crucial role that nuclear medicine can play in contemporary medicine and biomedical science.

Novel radiopharmaceutical applications in both diagnosis and therapy were presented, the time to make the transition to a lasting clinical reality is now! We should collaborate to pave the translational road, to bring promising novel tracers to (multicentre) clinical studies and reduce current hurdles, including legislation, high costs and reimbursement issues. “I Have a Dream, a Song to Sing”: we call upon all collaborators in nuclear medicine to join forces, to reduce internal competition, and to work on harmonized protocols in order to make promising new developments a clinical success both in diagnostics and therapy.
